# Charting the complete elastic properties of inorganic crystalline compounds

**DOI:** 10.1038/sdata.2015.9

**Published:** 2015-03-17

**Authors:** Maarten de Jong, Wei Chen, Thomas Angsten, Anubhav Jain, Randy Notestine, Anthony Gamst, Marcel Sluiter, Chaitanya Krishna Ande, Sybrand van der Zwaag, Jose J Plata, Cormac Toher, Stefano Curtarolo, Gerbrand Ceder, Kristin A. Persson, Mark Asta

**Affiliations:** 1 Department of Materials Science and Engineering, University of California, Berkeley, California 94720, USA; 2 Environmental Energy Technologies Division, Lawrence Berkeley National Laboratory, Berkeley, California 94720, USA; 3 Computational and Applied Statistics Laboratory, San Diego Supercomputer Center, University of California, San Diego, California 92093, USA; 4 Department of Materials Science 3ME, Delft University of Technology, Delft 2628CD, The Netherlands; 5 Department of Applied Physics, Eindhoven University of Technology, Eindhoven 5600MB, The Netherlands; 6 Department of Aerospace Engineering, Delft University of Technology, Delft 2629HS, The Netherlands; 7 Center for Materials Genomics, Department of Mechanical Engineering and Materials Science, Duke University, Durham, North Carolina 27708, USA; 8 Center for Materials Genomics, Materials Science, Electrical Engineering, Physics and Chemistry, Duke University, Durham, North Carolina 27708, USA; 9 Department of Materials Science and Engineering, Massachusetts Institute of Technology, 77 Massachusetts Avenue, Cambridge, Massachusetts 02139, USA

**Keywords:** Electronic structure, Metals and alloys, Computational methods, Computational chemistry

## Abstract

The elastic constant tensor of an inorganic compound provides a complete description of the response of the material to external stresses in the elastic limit. It thus provides fundamental insight into the nature of the bonding in the material, and it is known to correlate with many mechanical properties. Despite the importance of the elastic constant tensor, it has been measured for a very small fraction of all known inorganic compounds, a situation that limits the ability of materials scientists to develop new materials with targeted mechanical responses. To address this deficiency, we present here the largest database of calculated elastic properties for inorganic compounds to date. The database currently contains full elastic information for 1,181 inorganic compounds, and this number is growing steadily. The methods used to develop the database are described, as are results of tests that establish the accuracy of the data. In addition, we document the database format and describe the different ways it can be accessed and analyzed in efforts related to materials discovery and design.

## Background & Summary

The elastic tensor of a crystalline solid provides a complete description of its response to external forces within the elastic limit. This property is thus one of the most fundamental probes of the nature of the interatomic bonding in a given material system (e.g., ref. 1). Further, it is known that the elastic tensor correlates with many mechanical and thermal properties, and it is thus a critically important quantity for use in screening in the process of materials discovery and design. For example, since the work of Pugh in the 1950’s the ratio of the bulk to shear modulus has been used as a basis to understand and predict trends in the ductility of materials^2–9^. More recently, extensions of the Pugh analysis have been used to derive descriptors for hardness, in the attempt to discover new materials for hard coating applications, and to guide the search for the elusive inorganic compound with a hardness greater than diamond^3^. Elastic tensors can also be used to screen for materials with targeted thermal properties, as it provides a basis for rapid estimation of trends in heat capacities and thermal conductivities^10–13^. Knowledge of the full anisotropic elastic tensor can be used in conjunction with mathematical homogenization theories to predict the elastic response of composite materials, and thus guide the design of such materials with targeted stiffnesses^14,15^. Additionally, an area in which elastic properties find widespread use is geophysics, where acoustic velocities are used for interpretation of seismic data^16,17^.

Despite the importance of the elastic tensor, experimental data for this quantity is available for only a very small subset of all known inorganic compounds. This presents a fundamental bottleneck for the discovery and design of materials with targeted thermal and mechanical properties, or for performing continuum simulations of mechanical response that require elastic moduli as input. Considering only materials for which the full tensor of elastic coefficients is available, the classical works have references that sum up to a total number of around 150 independent systems for which experimental measurements have been compiled^18–26^. Considering papers that have investigated elastic constants of particular systems, this number might be twice as large, which is a very small fraction of the approximately 30,000 to 50,000 entries for ordered compounds in the inorganic crystal structure database^27–29^. Among the systems for which experimental data is available are approximately 70 pure elements, with the remainder consisting of binary systems and—to a much smaller extent—ternary systems and a variety of complex minerals. Among the binary materials are solid solutions and compounds, the latter often being ordered intermetallic compounds.

One challenge associated with using published experimental data for elastic moduli is that the spread in the reported values for a given system can be quite large, depending on the details of the experimental conditions and techniques employed. For example, elastic moduli derived from inelastic neutron scattering can be 10% greater than those derived from pulse-echo measurements^30^. Differences of over 20% in reported experimental values for the bulk and shear moduli for the same system have been observed in some cases, such as NiO^31,32^. Other experimental factors, such as different measurement temperatures^33^ and/or the presence of impurity phases, can also lead to variability in reported elastic constants.

Efforts aimed at developing databases of elastic moduli from first-principles computational methods have been undertaken in previous work (e.g., refs 34,35). Such a computational approach provides an advantage that all of the data can be derived in a consistent manner, facilitating comparisons across materials chemistries. In the present work we expand on this approach. Specifically, we present here the to-date largest database of calculated elastic properties of crystalline inorganic compounds, ranging from metals and metallic compounds to semiconductors and insulators. These calculations are part of a high-throughput (HT) effort^36^, undertaken within the framework of the Materials Project (MP) (www.materialsproject.org)^37^. The database of elastic tensors currently consists of over 1,181 materials and is being updated regularly. The elastic properties are obtained using first-principles quantum-mechanical calculations based on Density Functional Theory (DFT). As shown below, the calculated elastic constants are typically within 15% of experimental values, which represents a smaller scatter than that observed in experimental values in some cases. Pearson (*r*) and Spearman (ρ) coefficients indicate that the calculations performed in this work yield elastic properties that show an excellent correlation with experimental values, making the database presented here useful for screening materials with properties based on elastic tensors.

The remainder of the paper is organized as follows. We first describe our method for calculating elastic constants from DFT in a HT-environment. We then give an overview of the structure of the data, followed by a description of our results. Finally, we describe the verification and validation tests to assess the precision and accuracy of the chosen density functional and the HT algorithms employed in the calculations.

## Methods

### Generation of elasticity data

In this launch of the elastic constant database we tabulate results for a subset of 1,181 compounds chosen from those present in the current MP database. This subset includes 2 broad categories: i) metallic and small-band-gap compounds and ii) binary oxides and semiconductor compounds. The first category is taken from the MP-database, under the constraint that 1) the calculated bandgap <0.3 eV and 2) the energy above the convex hull (decomposition energy^38^) <0.5 eV/atom. These properties have been calculated previously by DFT using the standard HT-procedure and chosen MP parameters suitable for ground-state energy, lattice structure, and band structure^37,39^. The constraints are chosen to represent a set of materials that are metallic or near-metallic and energetically stable or near-stable, and yields the majority of the data set (approximately 1,100 systems). For the binary oxides, different selection criteria were used: 1) the bandgap >0.3 eV and 2) the energy above the convex hull=0 eV/atom, which yields approximately 100 systems. Furthermore, approximately 20 technologically relevant semiconductors were added to create a representative set of materials.

For these systems we compute the elastic constants using a stress-strain methodology. Specifically, starting from a relaxed structure for each compound, we generate a set of distorted structures, as follows. The Green-Lagrange strain tensor has 6 independent components, each of which is applied independently to every structure, with differing magnitudes, as described in the Workflow section below. For each deformed structure, the 3×3 stress tensor is calculated by DFT. If the components of the stress tensor are denoted by *S*_*ij*_ and the components of the Green-Lagrange strain tensor are denoted by *E*_*ij*_, the constitutive relation within linear elasticity can be written as in equation (1), which relates stresses to strains via the symmetric elastic matrix, with components *C*_*ij*_. In equation (1), the following Voigt-notation is employed: 11↦122↦233↦323↦413↦512↦6.

(1)[S11S22S33S23S13S12]=[C11C12C13C14C15C16C12C22C23C24C25C26C13C23C33C34C35C36C14C24C34C44C45C46C15C25C35C45C55C56C16C26C36C46C56C66][E11E22E332E232E132E12]

For each of the applied strains *E*_*ij*_, the full stress tensor is obtained from a DFT calculation in which ionic positions are relaxed. Consequently one row (or equivalently, column) of the elastic matrix is obtained from a linear fit of the calculated stresses over the range of imposed strains. Repeating this procedure for each of the 6 independent strain components, all elements of the elastic modulus tensor can be calculated. The result is a calculated set of *C*_*ij*_ values that can be used to calculate properties such as the bulk modulus *K* and the shear modulus *G*, as described in Table 1. The components of *C*_*ij*_ depend on the choice of coordinate system and lattice vectors, and in this work we have adopted the IEEE standard^40^ for all reported tensors.

The first-principles results presented in this work are performed using the projector augmented wave (PAW) method^41,42^ as implemented in the Vienna Ab Initio Simulation Package (VASP)^43,44^. In all calculations, we employ the Perdew, Becke and Ernzerhof (PBE) Generalized Gradient Approximation (GGA) for the exchange-correlation functional^45^. Other parameters employed in our HT-DFT calculations of elastic constants are system-dependent. For the metals and metallic compounds, we employ a cut-off for the plane waves of 700 eV. Further a uniform k-point density of approximately 7,000 per reciprocal atom (pra) is used, which means that the number of atoms per cell multiplied by the number of k-points equals approximately 7,000. For the compounds that contain magnetic elements, a ferromagnetic state is initialized in the calculation. We expect to correctly converge to ferromagnetic and non-magnetic states in this way, but not to anti-ferromagnetic states. This set of parameters results in elastic tensors that are converged to within 5% for 95% of the considered systems. Given the chemical breadth of the compound set—spanning metals, semiconductors and oxides—it is unlikely that one set of parameters performs equally well for all classes of materials. Therefore, to detect anomalies and outliers, tests were designed and corresponding first-principles calculations with higher convergence setting were performed (for more details see the next section). The set of approximately 20 semiconductors is calculated with the same convergence parameters as the metals and metallic compounds with similar resulting convergence. For the binary oxides, a plane wave cut-off of 700 eV is also used, with a k-point density of 1,000 pra. This leads to elastic constants converged to within 5% for all binary oxides considered in this work. Due to the presence of strongly correlated electrons in some of the oxides, the GGA+U method is employed, with U representing the Hubbard-parameter^46,47^. The values of U are chosen consistent with those employed in the MP^37,39^.

### Workflow

In this subsection we describe the workflow for the HT implementation of the stress-strain approach to computing elastic constants described above. We note that the workflow developed for this purpose shares many features in common with that developed for elastic constant-calculations in the Vlab distributed cyberinfrastructure for materials computation^34^. The main difference between the current approach and that described in ref. 34, is that the focus here is on elastic constants at zero pressure and temperature, whereas the Vlab workflow is developed more generally to consider elastic constants under finite pressures and temperatures, which are particularly important in the context of geophysical applications. The workflow in ref. 34 thus contains tasks related to the calculations of equations of state and finite-temperature phonon contributions, which are not considered in the present work. The emphasis here is on developing comprehensive databases of elastic moduli across a broad class of inorganic compounds, for materials design applications, and on interfacing the data with the Materials Project (MP) infrastructure.

**Figure 1:**
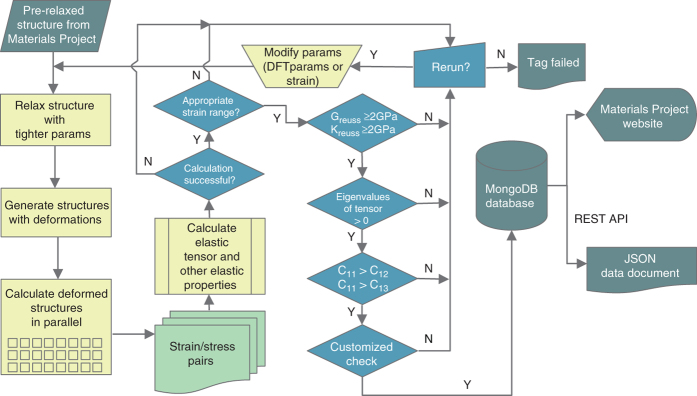
High-Throughput calculation scheme. Workflow for calculating and filtering the elastic constants.

Figure 1 summarizes the workflow for data generation implemented within the MP HT calculation infrastructure used in the present work. We start from the optimized structures in MP, and perform a tighter structural relaxation, with more stringent convergence parameters in the DFT-calculation. This initial step is necessary since the calculation of elastic constants by the stress-strain method requires a well-converged stress tensor, and the standard HT-settings in the MP, which are optimized for the total energy, are not always sufficient for this purpose. This procedure leads to a structure exhibiting close to zero residual stresses and forces on the atoms.

The next step is to construct a set of deformed structures, for calculations of the resulting stresses. Each structure in this set is deformed homogeneously by one of the 6 independent components of the strain tensor defined above, with a magnitude chosen over a prescribed range. Similar to previous work (e.g., refs 33,48) in which a stress-strain method for computing elastic tensors has been employed, a maximum strain of 1% is applied initially to distort the structures. In our experience this value is typically large enough for most compounds to minimize the numerical noise in the calculation of the stress tensor, but small enough to remain well within the linear-elastic regime. In this initial step we choose four values for the strain magnitude, varying between −1 to 1%, leading to a set of set of 24 initial deformed structures. First-principles calculations of the stress tensor for each of these structures are performed, including ionic relaxations. The calculation is considered to be unsuccessful if one or more of the single deformation runs fails to converge. In such cases, the calculations are rerun with tighter numerical convergence parameters. In the case of several unsuccessful iterations, a tag to this material is generated indicating that the calculation of the elastic tensor has failed.

Once the 24 stress tensor calculations have been successfully computed, a check is performed to determine whether the range of strains considered is appropriate for deriving the elastic constant tensor using a linear stress-strain relationship. This is done by fitting the elastic constants over different ranges of strain, and examining the sensitivity of the results. We employ the following nomenclature for the chosen ranges of strains investigated: *ε*_1_=(−1%, −0.5%, +0.5%, +1%), *ε*_2_=(−0.5%, +0.5%), *ε*_3_=(−0.75%, −0.5%, +0.5%, +0.75%), and finally *ε*_4_=(−1.25%, −0.75%, +0.75%, +1.25%). We first fit the elastic constants to the default strain range, *ε*_1_, and compare the resulting bulk and shear modulus to those as obtained from a fit to *ε*_2_. If the results are within 15%, we move on to the next step in the workflow using the elastic constants as obtained from the fit to the strain range *ε*_1_. If the discrepancies are larger than 15%, additional stress tensors are calculated for strain values of (±0.75%). We then compare the bulk and shear modulus, as fit from strain ranges *ε*_2_ and *ε*_3_. If the results agree to within 15%, we progress in the workflow using the elastic constants as fit to the strain range *ε*_2_. If again the results disagree, we compare the bulk and shear modulus, fit to *ε*_1_ and *ε*_4_. If these agree to within 15%, we progress in the workflow using the elastic constants fit to the default strain range *ε*_1_. If all of these steps fail, a warning message is generated for the compound, warranting further investigation.

From our initial set of 1,181 materials, we find that in 34 cases, either the bulk modulus and/or the shear modulus are different by over 15%, depending on whether *ε*_1_ or *ε*_2_ is used for fitting the elastic tensor. A refitting of the elastic constants of those systems is performed over the range of strains corresponding to *ε*_3_, and the bulk and shear moduli are compared to those as obtained from fitting to *ε*_2_. We find that only 20 systems exhibit discrepancies of over 15%. For the latter systems, we finally compare the bulk and shear moduli as obtained from fitting to *ε*_1_ and *ε*_4_, respectively, finding that only 10 still show discrepancies of over 15%. Thus, for the vast majority of the cases considered, the default range of strains *ε*_1_ is found to suffice for calculations of the elastic constants by a stress-strain methodology, and for more than two-thirds of the remaining compounds the additional checks implemented in the workflow lead to identification of an appropriate range of strains to yield reasonable results.

As illustrated in Fig. 1, for the systems where the calculation ends successfully and an appropriate range of strains is successfully identified, the elastic tensor results are further checked using various filters, designed to detect possible errors related to the assumption of linear elastic behavior, or other numerical inaccuracies that might occur due to the need for tighter convergence. The filters are designed to reveal physically unlikely behavior or mechanical instabilities behavior, which can be indicative of such errors. The filters include: i) *K*_*R*_ >2 GPa, ii) *G*_*R*_ >2 GPa, iii) all 6 eigenvalues of the elastic tensor are larger than zero, and iv) Born-Huang stability criteria^49^ are obeyed to within a 10% margin (see below). Note that *K*_*R*_ and *G*_*R*_ represent the Reuss-averages of the bulk and shear moduli, respectively^50^ (see Table 1 for definitions).

Conditions i) and ii) are selected based on an empirical observation that the most compliant known metals have shear and bulk moduli larger than approximately 2 GPa. Hence if our calculations yield results below 2 GPa for either the Reuss averages^50^ (a lower bound estimate) of *K* or *G*, these results might be correct but deserve additional attention. Condition iii) expresses the conditions for mechanical stability of solids under zero stress. If one (or more) of the eigenvalues of the elastic tensor is (are) negative, the compound is mechanically unstable at zero temperature. The effects of finite temperatures may lift the mechanical instability in some systems, such as B2 NiTi^51^. However, negative eigenvalues may also indicate the calculation is erroneous, and hence these cases are flagged for a more detailed investigation. The final set of filters iv) is used to identify elastic tensors that correspond to materials that are mechanically stable but are near an elastic instability. This is done by applying the Born-Huang elastic stability criteria for the appropriate crystal system. As an example for the cubic crystal system, we require that *C*_11_−*C*_12_>0, *C*_11_+2*C*_12_>0, *C*_44_>0. If one or more of these criteria is violated, one or more of the elastic tensor eigenvalues is negative. To identify compounds that are close to a mechanical instability, we apply a small tolerance to the Born-Huang criteria. As an example, for the case of cubic crystal systems, we check if *C*_11_>*ϵC*_12_ holds true, where *ϵ*=1.1. We find empirically that when *C*_11_<*ϵC*_12_, frequently the first-principles calculation was not properly converged or a more accurate PAW potential is required (e.g., including semi-core states). For other crystal systems, similar tests are performed.

For the materials that do not obey one or more of the conditions i)-iv), we investigate the effect of the various convergence parameters in the DFT calculations, and if the results still do not pass the filters, a warning tag is generated warranting further investigation. From the initial set of 1,181 materials, it is found that 97 systems fail to meet criteria i)-iv). In particular, 57 systems are found to be mechanically unstable, 16 systems have Reuss averaged shear or bulk moduli lower than 2 GPa and 19 systems are within a margin *ϵ*=1.1 of being mechanically unstable. For these 97 systems, a new set of calculations is performed using a substantially higher k-point density of approximately 25,000 pra in both structural relaxations and stress-calculations. This set of calculations results in a reduction in the number of systems that do not obey conditions i)—iv) from 97 to 76 systems. Of these, 50 systems are found to be mechanically unstable, 14 systems have Reuss averaged shear or bulk moduli lower than 2 GPa and 12 systems are mechanically stable but within a margin *ϵ*=1.1 of being mechanically unstable. In particular, the pure metals Al and Cu are flagged by the filters in the initial DFT-runs employing lower k-points, since these metals are close to mechanical instability. However, upon increasing the k-points, results improve (this finding was not unexpected since Cu and Al which are known to exhibit complex Fermi surfaces^52^). The filters described above are designed to identify anomalies, and they will likely be refined as our approach evolves and additional validation is performed.

All elastic tensors that have achieved sufficient numerical convergence are inserted into the MP database and reported on the web site. We also store and report on the website results for mechanically unstable compounds, but include a warning message to the user. A JSON (JavaScript Object Notation) data document is generated for each reported elastic tensor. This JSON data document is publicly available at the Dryad-repository (Data Citation 1). We perform the structure generation and data analysis for elastic constant calculations using our open-source materials analysis code pymatgen^53^. The workflow software FireWorks^54^ is used to automate the HT calculations and data management.

### Code availability

The code for calculating elastic constants and related properties is part of the open-source code pymatgen^53^. Pymatgen is released under the MIT (Massachusetts Institute of Technology) License and is freely accessible. The workflow as shown in Fig. 1 is powered by the open-source code FireWorks and is released under a modified GPL (GNU General Public License). Also FireWorks can be accessed and used freely.

## Data Records

The calculated elastic property data and related metadata of 1,181 materials are publicly available at the Materials Project (www.materialsproject.org). The complete data set can be downloaded in a JSON (Data Citation 1) file or via the Materials Project REST API. The Materials Project also provides a convenient web interface that allows searching for materials with particular properties by querying the elastic constant database. In addition, the materials detail pages on the website now include calculated elasticity data when available.

### File format

The data set for each material is stored as an individual JSON document (Data Citation 1). Based on a series of key/value pairs, the JSON format offers a readily parsable yet human readable solution for data exchange. The metadata record for each material includes descriptions of the material (e.g., structure, structure symmetry) and calculation parameters (e.g., k-points density). The JSON keys for the metadata and their descriptions are listed in Table 2. Note that the structure is presented both in Crystallographic Information File (cif) and poscar-format. The poscar-format is the standard structure description used by the VASP-code.

### Properties

The elastic constants appearing in equation (1) are calculated by DFT and represent the elastic constants of a single crystal. While single-crystal elastic properties are important as input into higher length-scale modeling of mechanical behavior, we also derive and report several polycrystalline averaged properties. In this work, we calculate for all considered systems the Voigt and Reuss averages of the bulk and shear modulus. The Voigt average provides an upper bound on the elastic moduli of an untextured polycrystalline material whereas the Reuss average provides a lower bound^50^. The experimental quantities will lie between the bounds, with the precise value determined by the detailed orientation of the various grains in the material. Also we provide the empirical VRH-average for the bulk and shear modulus. This empirical average is known to represent the bulk and shear modulus of polycrystalline materials with comparable accuracy as more advanced polycrystalline homogenization schemes such as those by Hashin and Strickman^14,55^. Other properties computed in this work are the index of elastic anisotropy^56^ and the Poisson ratio in the isotropic approximation. The various derived properties are listed in Table 1, including expressions relating these properties to the elements of the single-crystal elastic tensor. The corresponding JSON keys and the datatypes are also listed in Table 1. The elastic tensor *C*_*ij*_ is presented in two ways in Table 1: i) in the standardized IEEE-format and ii) in the format corresponding to the orientation of the crystal structure as defined in the poscar-key in Table 2.

### Graphical representation of results

**Figure 2:**
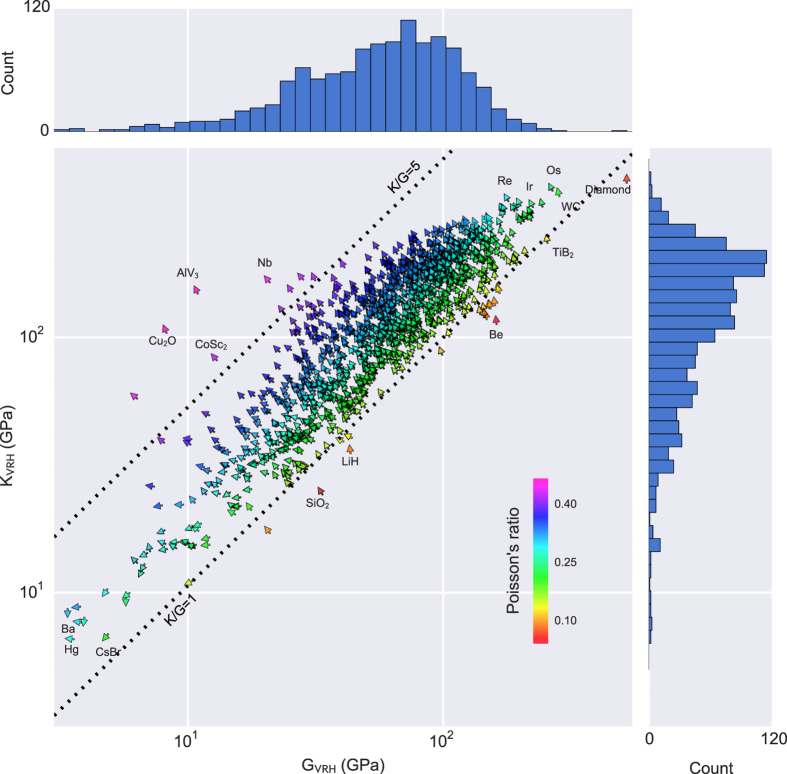
Distribution of calculated volume per atom, Poisson ratio, bulk modulus and shear modulus. Vector field-plot showing the distribution of the bulk and shear modulus, Poisson ratio and atomic volume for 1,181 metals, compounds and non-metals. Arrows pointing at 12 o’clock correspond to minimum volume-per-atom and move anti-clockwise in the direction of maximum volume-per-atom, which is located at 6 o’clock. Bar plots indicate the distribution of materials in terms of their shear and bulk moduli.

A graphical representation of our dataset is presented in Fig. 2, which shows a log-log plot of the VRH averaged bulk modulus versus the VRH averaged shear modulus for all materials considered in this work. The orientation of each arrow corresponds to the volume per atom (VPA) of that specific material. The material with the minimum VPA in our dataset is assigned an arrow pointing at 12 o’clock (diamond) and the arrows rotate anti-clockwise towards the materials with the maximum VPA in our dataset at 6 o’clock (barium). The angle of rotation from 12 o’clock to 6 o’clock is proportional to the normalized VPA. The VPA is considered since it is known to correlate well with elastic properties such as bulk modulus^57–59^. Indeed, Fig. 2 illustrates this apparent correlation. Specifically, diamond exhibits the highest bulk and shear moduli of all materials in our database and it also has the smallest VPA among those materials. The more elastically compliant materials in Fig. 2 show relatively higher values for the VPA. The color coding in Fig. 2 represents the Poisson ratio in the isotropic approximation. Also, two lines of constants *K*_*VRH*_/*G*_*VRH*_ ratio are drawn. As described in the Introduction, this quantity, known as Pugh’s ratio^2^, has been shown to correlate with ductility in crystalline compounds^2,3^ and is further related to the Poisson ratio^5^. The bar plots show the distribution of materials relative to their respective values for the bulk and shear modulus. The distribution shows that most materials considered, lie in the region around 80 and 190 GPa for the shear modulus and bulk moduli, respectively. Thus, this diagram distills several well-known results in the field of elasticity and illustrates them through a large amount of data.

## Technical Validation

### Verification of computational methodology

To verify proper implementation of HT version of the stress-strain method described above, detailed comparisons have been undertaken between the data derived from this approach and independent computational results obtained in the present work using alternative methods, or published previously by other authors using the same DFT approximations. Such comparisons have been undertaken for a subset of systems that are representative of the material types in the database. Overall, the comparisons yield agreement at the level of approximately 5%, with a few exceptions, as described below.

Considering first insulator compounds, the *C*_*ij*_ values obtained here for *α*-Al_2_O_3_ are all within 2% of the results reported in ref. 33 using the same DFT approximations, combined with a similar stress-strain method. The present *α*-Al_2_O_3_ results are also within 3% of the values for all *C*_*ij*_ components obtained from a numerical differentiation of the energy versus strain using Wien2K^60,61^, and within 5% of the results for all components obtained by energy differentiation methods derived from Quantum Espresso^62^ and reported in ref. 63. It should be noted that for *α*-Al_2_O_3_ the *C*_14_ component obtained in this work has a sign opposite to that reported in ref. 63. In fact, the sign of *C*_14_ in *α*-Al_2_O_3_ has been a source of controversy in other previous theoretical and experimental studies^33,63,64^. However, as discussed in ref. 63, the ambiguity in choosing the Cartesian reference coordinate system for trigonal materials with R centering type is the likely cause of these discrepancies. For cubic Y_2_O_3_ the present results for each of the *C*_*ij*_ components are within 10% of those reported from the stress-strain calculations performed within GGA in ref. 64 (the largest discrepancy is found for the *C*_12_ component). For *β*-Si_3_N_4_ the present results agree to within 5% of those reported in the same publication^64^. For the polar wurtzite ZnO compound, the results obtained in the present work agree to within 8% for *C*_44_, and within 2% for all other moduli, with the values obtained by Wu *et al.* ^65^ using the same DFT approximations, and an approach that employs density-functional perturbation theory to compute internal displacement contributions.

We have also conducted a number of comparisons between the present results and other theoretical calculations for metallic and small-band-gap systems. We have compared results obtained using our HT methodology with those derived from a method that fits the calculated total energy as a function of volume-conserving strains, as developed by Mehl *et al.* ^48,66^. The present HT stress-strain methodology yields results within 4% of those obtained from this energy versus strain method for BCC Lithium and FCC Aluminum. Further, the elastic constant tensor components for orthorhombic TiB, reported from full-potential-linear-augmented-plane-wave GGA calculations, along with total energy differentiation methods^67^, are within 5% for of the values obtained here for all *C*_*ij*_ components, with the exception of *C*_44_ (reported as *C*_66_ in ref. 67), which is within 15%.

As described in the previous section, consistency checks are built into the HT-workflow employed in the present work to ensure that the range of strains employed in the fit of the stress-strain relations are appropriate. The dependence of calculated elastic constants on the range of strains considered has been examined in detail in previous work, e.g., ref. 63. The authors of ref. 63 employ an energy versus strain method, using sixth-order polynomial fits of the energy to a strain range of up to 8%. The authors conclude that for small deformations, the best results are obtained by low-order polynomial fits, and that the stress-strain approach is more accurate in the sense that only first-order derivatives are required, in which case smaller distortions are required. This is consistent with the findings in this work, where a maximum strain of 1% is found to provide reliable results for over 97% of the compounds considered, using a linear stress-strain fit. To investigate this issue further we have performed detailed tests similar to those in ref. 63 for a select number of systems. Strains in the range of 1 to 8% were applied and the stresses and strains fit using *n*-th order polynomials, where *n* ranges from 1 to 4. In particular for KBr, which is one of the most elastically compliant materials in the database, we found changes of less than 2% in the bulk and shear moduli, as the strain was varied from 1 to 8%, regardless of the order of the polynomial. For diamond, the stiffest material in our database, one might expect relatively strong non-linear behavior of the stress with strain, even for small strains. However, also for diamond we find that the bulk and shear moduli vary by less than 2% as the strain is varied and the polynomial order ranges from 1 to 4. Overall, the tests described in this and the previous section suggest that the stress-strain approach and the range of strains considered in its application, yield reliable results for the vast majority of the compounds considered in the development of the current database.

### Validation through comparison to experimental measurements

A comprehensive literature review was performed to compile measured elastic constant tensors, for comparison with the present calculations, in order to establish the expected accuracy of the calculated results. In this comparison we consider only experimental sources that report the full elastic tensor, rather than only the bulk or shear modulus, so that a systematic comparison with the calculated elastic tensors can be made. In total, 104 systems are used in the comparison, including oxides and semiconductors^18,20,23,30,32,68–74^ and metals and metallic compounds^18,20,22,26,75–79^. In the comparison, we make use of the Voigt-Reuss-Hill average for *K* and *G* (denoted by *K*_*VRH*_ and *G*_*VRH*_, respectively), which is the arithmetic mean of the Voigt and Reuss bounds^50^. See also Table 1 for their definitions. The shear (*G*_*VRH*_) and bulk (*K*_*VRH*_) moduli of these 104 systems are compared by calculating the VRH-average from the experimentally measured and calculated tensors. In addition a Euclidean difference norm^80^, normalized by the magnitude of the calculated elastic tensor, is used to probe errors relative to the mean elastic constants: $\left\|C_{ij}^{exp}-C_{ij}^{calc}\right\|\cdot {\left\|C_{ij}^{calc}\right\|}^{-1}$${\left\|C_{ij}\right\|}_{E}={\left(\mathrm{tr}\left[C_{ij}^{T}C_{ij}\right]\right)}^{0.5}$. In this expression, *C*_*ij*_ represents the elastic tensor (in matrix form) as defined in equation (1).

**Figure 3:**
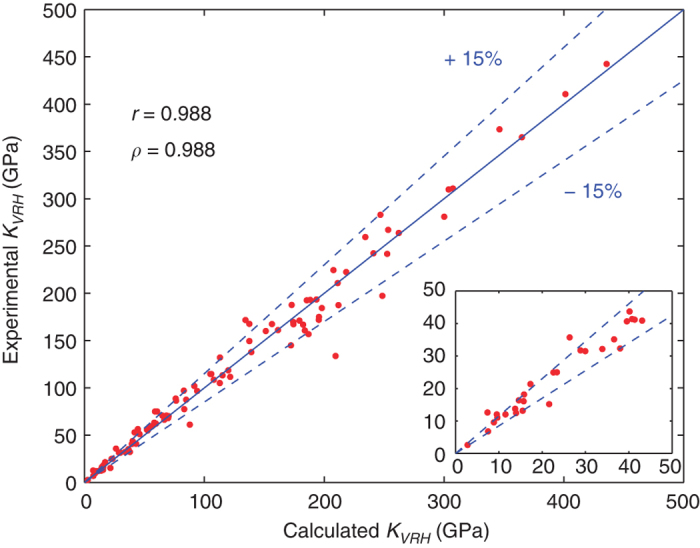
Plot of experimental versus calculated bulk moduli. Comparison of experimental and calculated bulk moduli for a selected set of systems, with calculated Pearson correlation coefficient *r* and Spearman correlation coefficient ρ reported.

**Figure 4:**
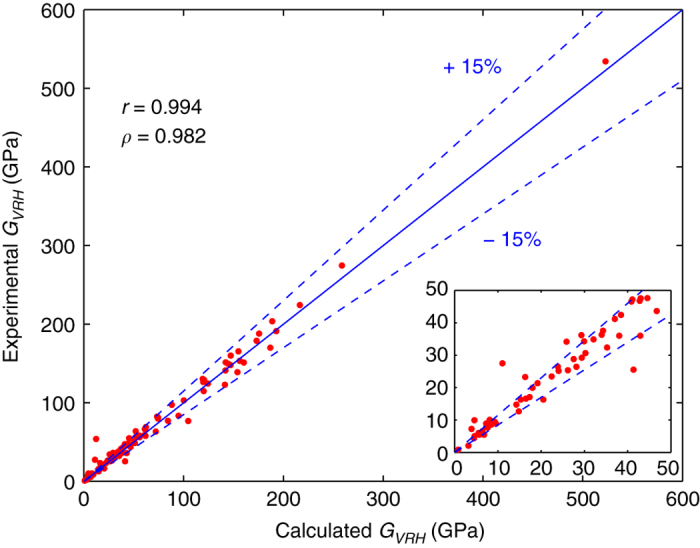
Plot of experimental versus calculated shear moduli. Comparison of experimental and calculated shear moduli for a selected set of systems, with calculated Pearson correlation coefficient *r* and Spearman correlation coefficient ρ reported.

The comparison of calculated and experimental values for *K*_*VRH*_ and *G*_*VRH*_ are shown in Figs 3 and 4, respectively. In each plot, lines are shown indicating relative differences between computation and experiment of ±15%. As can be seen, the agreement between experiment and calculation is generally within this threshold, although there are some outliers. Specifically, in the case of the bulk modulus a discrepancy between experiment and calculations larger than 15% is found for 16 systems (in order of absolute deviation, from low to high): Na, Tl, Pb, Ca, CsI, Nd, Yb, YZn, Cd, Mg_2_Sn, Ge, Pt, CaAl_2_, Au, Co, CdAu. The first 11 in the list disagree with experiment by less than 10 GPa. For the shear modulus, a discrepancy between experiment and calculations larger than 15% is found for 15 systems (in order of absolute deviation, from low to high): KI, Ca, CsI, KBr, CdSe, Tl, Cd, GaSb, GaAs, Ge, CdAu, Y_2_O_3_, Au, Cr_3_Si, MnSi. The first 6 in the list disagree with experiment by less than 10 GPa. These larger discrepancies may be due to errors in the calculations, the experimental measurements or a combination of both. Note that most of the systems displaying greater than 15% discrepancy between calculations and measurements are those with relatively low bulk and shear moduli, see the insets in Figs 3 and 4. Similarly, we find for the quantity $\left\|C_{ij}^{exp}-C_{ij}^{calc}\right\|\cdot {\left\|C_{ij}^{calc}\right\|}^{-1}$ most of the systems show discrepancies below 20%, with the largest discrepancies found for the systems with the smallest values of $\left\|C_{ij}^{calc}\right\|$. For these systems with relatively small elastic moduli, the discrepancies may be due to the larger effect of the numerical errors in the calculations on the relative precision of the calculated elastic tensors.

Other factors that might contribute to discrepancies are temperature variations: DFT provides a zero-temperature description of the state of the material, whereas many experiments are done at room temperature. While such temperature variations are typically relatively small below room temperature, in some systems this effect can be large. For example, in previous experimental studies of single-crystal Nb_3_Sn, the value of (*C*_11_-*C*_12_) starts at 140 GPa and decreases to zero as temperature decreases from 300 to 32 K ref. 75. Our calculated results for Nb_3_Sn at 0 K show a mechanical instability with *C*_11_ slightly less than *C*_12_. Thus, these mechanical instabilities can contain useful information indicating potentially anomalous mechanical properties or shear instabilities at low temperature. Methods have been implemented in the literature to predict the temperature dependence of the elastic constants from first-principles^81,82^, and implementation of such approaches represents a future extension of the database. The elastic constants reported in this work represent the zero-temperature limit of the isothermal moduli, whereas experimentally it is often the adiabatic elastic tensor that is measured; however, the differences between these two types of elastic constants are typically small^83^. From the computational perspective, we have found that for some elements, PAW potentials exhibiting a different number of electrons as valence states can significantly affect the calculated elastic properties. This is the case for the elements V, Ti and Nb. Also, some of the systems listed above exhibit antiferromagnetic states. These states are both temperature and strain dependent, and resolving these details in HT DFT-calculations of elastic constants is challenging and the topic of current work that is expected to impact future releases of the database.

For the purpose of using the elastic constant database in the context of materials discovery, it is useful to characterize the correlation between the calculated and measured elastic quantities. For this purpose we again consider the values for *K*_*VRH*_ and *G*_*VRH*_, and calculate the Pearson and Spearman correlation coefficients (*r* and *ρ*, respectively). Also computed are 95% bootstrap-based confidence intervals for the correlations. The lower (LB) and upper (UB) bounds of these confidence intervals are presented as ([LB, UB]). For the bulk modulus, the Pearson and Spearman correlation coefficients are 0.988 ([0.978,0.994]) and 0.988 ([0.973,0.993]), respectively. For the shear modulus, we find values of 0.994 ([0.985,0.998]) and 0.982 ([0.955,0.993]) for the Pearson and Spearman correlation coefficients, respectively. These values suggest that the measured and calculated values for bulk and shear moduli are strongly linearly associated and also, a high monotone association exists.

## Usage Notes

The database presented here represents the to-date largest collection of consistently calculated or measured elastic tensors for crystalline inorganic materials. We anticipate that this dataset, and the methods provided for querying it, will provide a useful tool in fundamental and application-related studies of inorganic compounds. We expect, in particular, that the database will be useful for efforts aimed at materials discovery and design, in the search for and optimization of materials with targeted mechanical and thermal properties. For the first time, researchers will be able to query existing compounds from the database by specifying desired elastic properties, for example a maximum value of the shear modulus with minimum elastic anisotropy. For compounds that are currently not in the database, future extensions of this work will be a web interface where MP-users will be able to calculate elastic properties on demand, by uploading a file describing the crystallography of the material of interest. Techniques such as data mining and machine learning can be used to reveal fundamental trends in the elastic properties of compounds, and guide the screening of potentially interesting materials for target properties.

## Additional information

**How to cite this article:** de Jong, M. *et al.* Charting the complete elastic properties of inorganic crystalline compounds. *Sci. Data* 2:150009 doi: 10.1038/sdata.2015.9 (2015).

## Supplementary Material



## Figures and Tables

**Table 1:** Properties derived from the elastic constant matrix in this work, and their corresponding JSON keys and datatypes.

**Property**	**Key**	**Datatype**	**Unit**	**Description**	**Equation**
Elastic tensor, *C*_*ij*_	elastic_tensor	array	GPa	Tensor, describing elastic behavior (IEEE-format)	see main text
Elastic tensor, *C*_*ij*_	elastic_tensor_original	array	GPa	Tensor, describing elastic behavior, corresponding to poscar orientation	see main text
Compliance tensor, *s*_*ij*_	compliance_tensor	array	GPa^−1^	Tensor, describing elastic behavior	$s_{ij}=C_{ij}^{-1}$
Bulk modulus Voigt average, *K*_*V*_	K_Voigt	number	GPa	Upper bound on *K* for polycrystalline material	9*K*_*V*_=(*C*_11_+*C*_22_+*C*_33_) +2(*C*_12_+*C*_23_+*C*_31_)
Bulk modulus Reuss average, *K*_*R*_	K_Reuss	number	GPa	Lower bound on *K* for polycrystalline material	1/*K*_*R*_=(*s*_11_+*s*_22_+*s*_33_) +2(*s*_12_+*s*_23_+*s*_31_)
Shear modulus Voigt average, *G*_*V*_	G_Voigt	number	GPa	Upper bound on *G* for polycrystalline material	15*G*_*V*_=(*C*_11_+*C*_22_+*C*_33_) −(*C*_12_+*C*_23_ + *C*_31_) +3(*C*_44_+*C*_55_+*C*_66_)
Shear modulus Reuss average, *G*_*R*_	G_Reuss	number	GPa	Lower bound on *G* for polycrystalline material	15/*G*_*R*_=4(*s*_11_+*s*_22_+*s*_33_) −4(*s*_12_+*s*_23_+*s*_31_) +3(*s*_44_+*s*_55_+*s*_66_)
Bulk modulus VRH average, *K*_*VRH*_	K_VRH	number	GPa	Average of *K*_*R*_ and *K*_*V*_	2*K*_*VRH*_=(*K*_*V*_+*K*_*R*_)
Shear modulus VRH average, *G*_*VRH*_	G_VRH	number	GPa	Average of *G*_*R*_ and *G*_*V*_	2*G*_*VRH*_=(*G*_*V*_+*G*_*R*_)
Universal elastic anisotropy, *A* ^*U*^	elastic_anisotropy	number	—	Description of elastic anisotropy	*A* ^*U*^=5(*G*_*V*_/*G*_*R*_) +(*K*_*V*_/*K*_*R*_) −6≥0
Isotropic Poisson ratio, *μ*	poisson_ratio	number	—	Number, describing lateral response to loading	*μ*=(3*K*_*VRH*_−2*G*_*VRH*_)/(6*K*_*VRH*_+2*G*_*VRH*_)

**Table 2:** JSON keys for metadata and their descriptions.

**Key**	**Datatype**	**Description**
material_id	string	IDs for entries in the Materials Project
formula	string	Chemical formula
structure	string	Relaxed crystal structure represented in Crystallographic Information File (cif)
poscar	string	relaxed crystal structure represented in poscar-format for VASP calculations
space_group	number	Space group number defined by The International Union of Crystallography
volume	number	Volume of the relaxed structure in Å^3^
nsites	number	Number of atomic sites for the conventional cell
kpoint_density	number	density of k-points in the first Brillouin zone per reciprocal atom
